# The Synthesis Process and Thermal Stability of V_2_C MXene

**DOI:** 10.3390/ma11112112

**Published:** 2018-10-27

**Authors:** Meng Wu, Bingxin Wang, Qianku Hu, Libo Wang, Aiguo Zhou

**Affiliations:** 1 School of Materials Science and Engineering, Henan Polytechnic University, Jiaozuo 454000, Henan, China; wumeng32323@163.com (M.W.); wbx0816@126.com (B.W.); hqk@hpu.edu.cn (Q.H.); wanglibo537@hpu.edu.cn (L.W.); 2 Henan International Joint Research Laboratory for High-Performance Light Metallic Materials and Numerical Simulations, Henan Polytechnic University, Jiaozuo 454000, Henan, China

**Keywords:** MXene, V_2_C, synthesis, thermal stability

## Abstract

The effect of etching solution on the synthesis process of two-dimensional vanadium carbide (V_2_C MXene) was researched. Three etching solutions were used to etch ternary carbide V_2_AlC at 90 °C. The three solutions were: lithium fluoride + hydrochloric acid (LiF + HCl), sodium fluoride + hydrochloric acid (LiF + HCl), and potassium fluoride + hydrochloric acid (KF + HCl). It was found that only NaF + HCl solution was effective for synthesizing highly pure V_2_C MXene. The existence of sodium (Na^+^) and chloridion (Cl^−^) in etching solution was essential for the synthesis. The thermal stability of the as-prepared V_2_C MXene in argon or air was studied. From thermogravimetry and differential thermal analysis, V_2_C MXene was found to be stable in argon atmosphere at a temperature of up to 375 °C. As the temperature increased, V_2_C MXene was gradually oxidized to form nanoparticles composed of vanadium trioxide (V_2_O_3_) and a part of V_2_C MXene was broken and transformed to vanadium carbide (V_8_C_7_) at 1000 °C. In air atmosphere, V_2_C MXene was stable at 150 °C. At 1000 °C, V_2_C MXene was oxidized to form vanadium pentoxide (V_2_O_5_).

## 1. Introduction

In 2011, a novel two-dimensional (2D) transition metal carbide, Ti_3_C_2_, was synthesized by removing the Al layer from titanium aluminum carbide (Ti_3_AlC_2_) in hydrofluoric acid (HF) [1]. The precursor, Ti_3_AlC_2_, is a member of MAX phases, which is a family of ternary carbide or nitride with the formula M_n+1_AX_n_, where M is a transition metal, A is the main-group element, and X is either or both C and N. Inspired by the synthesis of Ti_3_C_2_, similar 2D materials, such as Ti_2_C [2], Mo_2_C [3], Nb_2_C, and V_2_C [4], were synthesized. These 2D materials are made by removing the A element from MAX phases and have a similar structure to graphene, and are thus named MXenes. Because MXenes are made in aqueous solution of F^−^, due to the high surface energy, the surface of MXenes are always terminated with F/OH/O groups [5,6,7]. T_x_ was used to denote the surface terminated functional groups. Thus, the chemical formula of Ti_3_C_2_ can be changed to Ti_3_C_2_T_x_, and that of V_2_C can be changed to V_2_CT_x_.

MXenes havemany important applications in many fields, such as in the anode of a lithium-ion battery (LIB) [4,8,9,10,11] and the catalyst for the hydrogen evolution reaction (HER) [3,12,13]. Recently, based on theoretical calculation [14], V_2_C MXene was found to have a better performance as an anode of LIB than many other MXenes. Additionally, many applications of V_2_C MXene have been reported. V_2_C MXene can be used as the positive electrode of sodium ion capacitors, showing good results for sodium ion capacitors [15]. V_2_C MXene was reported to be used to capture CO_2_ in the interlayer space [16]. Furthermore, it was also found that V_2_C MXene decorated with metals displays extremely high catalytic activity for the hydrogen evolution reaction (HER), which provides a new possibility for cost-effective alternatives to the noble metal Pt [6].

Although V_2_C MXene theoretically has a much better performance than other MXenes, highly pure V_2_C MXene is difficult to be made. This is because the formation energy of V_2_C MXene is lower than that of other MXenes [17]. The common method employed to make MXenes has been to etch MAX phases precursor in HF solution at room temperature [1,5,18]. However, V_2_C MXene made by this method has been shown to contain plenty of vanadium aluminum carbide (V_2_AlC) precursor [4,15,19,20].

In 2017, we found a new method, sodium fluoride/hydrochloric acid (NaF + HCl) etching at 90 °C, to synthesize V_2_C MXene. By that method, highly pure V_2_C MXene with good Li storage properties was synthesized [21]. This work was inspired by a report on synthesizing Ti_3_C_2_ MXene by lithium fluoride/hydrochloric acid (LiF + HCl) etching [22]. Thus, a question was proposed. Why is NaF+HCl rather than LiF+HCl a suitable etchant? If NaF is replaced by LiF or other fluorides, can V_2_C be synthesized? One of the purposes of this paper is to answer this question. Three fluorides were used to make etching solutions with HCl for V_2_C MXene synthesis. The effects of these solutions on the etching process were clarified.

Because of the low formation energy, V_2_C MXene is difficult to synthesize. For the same reason, V_2_C MXene is highly unstable. Li et al., studied the thermal stability of Ti_3_C_2_ in oxygen and argon [23]. Zhou et al., studied the structural stability of Zr_3_C_2_ and Ti_3_C_2_ at elevated temperatures. Compared to Ti-based MXene, Zr_3_C_2_ exhibits relatively better high-temperature stability [24]. As the authors know, there is no report on the thermal stability of V_2_C MXene. Therefore, another purpose of this paper is to study the thermal stability of V_2_C MXene by thermal analysis.

## 2. Experimental

### 2.1. Syntheses of V_2_C MXene

V_2_C MXene was produced by immersing V_2_AlC powders in etching solution. The V_2_AlC powders were synthesized by the molten salt method [25] from vanadium powders (V, 99.6 wt.%, 325 mesh, Xingrongyuan Company, Beijing, China), aluminum powders (Al, 99.6 wt.%, 200 mesh, Xingrongyuan Company, Beijing, China), and graphite powders (C, 99.0 wt.%, 200 mesh, Jingchunshenghua Company, Shanghai, China). V, Al, and C powders were weighed according to the molar ratio of 2:1.2:1. Then, sodium chloride (NaCl) powders (99.5 wt.%, Yongda Company, Tianjin, China) were weighed according to the mass ratio of (2V/1.2Al/C): NaCl = 1:1. All powders were mixed by a ball mill machine for 12 h. Then, the mixtures of 2V/1.2Al/C and NaCl were placed in an alumina crucible and put in a tube furnace. The samples were annealed in flowing Ar atmosphere (99%) at 1400 °C for 2 h. The obtained sample was crushed and sieved through a 500-mesh sieve to yield powders.

The etching solution was made by mixing fluoride with hydrochloric acid (HCl, 6 mol/L, Shuangshuang Chemical Company, Yantai, China). The fluoride was NaF (≥98 wt.%, Sinopharm Chemical Reagent Company, Beijing, China), LiF (≥98.5 wt.%, Guangfu Fine Chemical Research Institute, Tianjin, China), or KF (≥99 wt.%, Chemical Reagent Factory, Luoyang, China). The fluoride (2.00 g NaF, 1.24 g LiF or 4.48 g KF·2H_2_O) was ultrasonic mixed with 40 mL HCl and 40 mL distilled water for 15 min, respectively. The mass difference of fluoride was used to obtain etching solution with the same molar concentration (0.6 mol/L). After that, 1.44 g V_2_AlC powders were immersed in the solutions, and the solutions were then kept for 72 h at 90 °C with magnetic stirring. Thereafter, V_2_C MXene powders were centrifugally separated from the etching solutions, and washed with deionized water and ethanol repeatedly to remove possible absorbed ions and remaining precursors. Before characterization, the obtained powders were dried in vacuum at 80 °C for 24 h.

The abbreviations and the corresponding full names of chemical substances that appear in this paper are listed in Table 1.

### 2.2. Characterization

X-ray diffraction patterns of sample powders were obtained with an X-ray diffractometer (XRD; Rigaku, Samart-lab, Tokyo, Japan) with Cu K_α_ radiation, λ = 1.5406 Å. A field emission scanning electron microscope (SEM; Merlin Compact, Carl Zeiss NTS GmbH, Jena, Germany) equipped with an energy disperse spectroscope (EDS; X-Max^N^, Oxford, UK) and a transmission electron microscope (TEM; JEOLJEM-2010, Tokyo, Japan) with an accelerating voltage of 200 kV were used to observe the microstructure morphology and element distribution of samples. Raman spectrum was recorded with a confocal spectrometer (Horiba JobinYvon, LabRAM HR800, Paris, France), using the 514.5 nm excitation of the argon laser at room temperature. The Raman spectral resolution was <0.35 cm^−1^.

The thermal stability of the sample was analyzed by a thermal analyzer (STA449C, Netzsch, Selb, Germany) with α-Al_2_O_3_ pans under argon/air flow with a heating rate of 5 °C/min from room temperature (RT) to 1000 °C. The thermogravimetric (TG) curve and differential thermal analysis (DTA) curve were obtained by this analysis.

## 3. Results and Discussion

### 3.1. Synthesis Process Analysis

**Figure 1:**
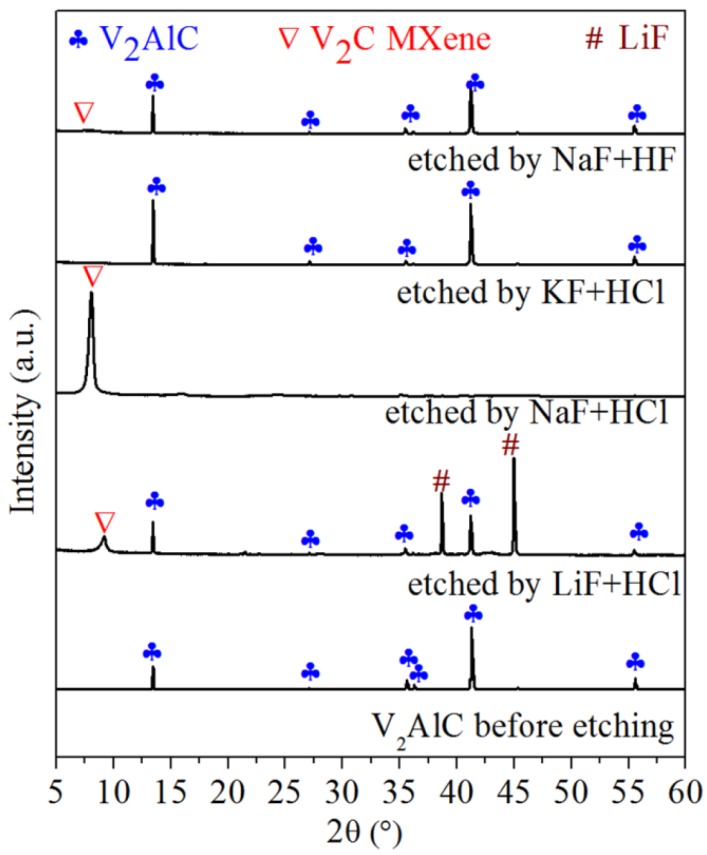
XRD patterns of V_2_AlC samples before and after the etching in different solutions.

The XRD patterns of V_2_AlC samples before and after the etching in different solutions are shown in Figure 1.

In Figure 1, after V_2_AlC powders are etched by LiF + HCl for 72 h, the peaks of V_2_AlC still exist and very strong diffraction peaks of LiF appear. A weak peak appears at 2θ ≈ 9°, which belongs to newly formed V_2_C MXene. However, the main diffraction peaks still belong to V_2_AlC and LiF. Thus, LiF + HCl etching cannot make highly pure V_2_C MXene. After etching in NaF + HCl solution, the diffraction peaks of V_2_AlC completely disappear and a strong peak at 8.03° appears, corresponding to the (002) plane of V_2_C MXene. This result indicates that Al was selectively etched off from V_2_AlC and most V_2_AlC had already been transformed to V_2_C MXene. The *c* lattice parameter (*c*-LP) of V_2_C calculated from the 2θ is 22.0 Å. The c-LP of V_2_C MXene made by HF etching at RT was 19.8 Å [15]. Compared with that V_2_C MXene, the V_2_C made by this method has obviously larger *c*-LP and a higher purity. Thus, highly pure V_2_C MXene can be made by NaF + HCl etching. However, after etching in KF + HCl solution, there were only the diffraction peaks of V_2_AlC in the product; in other words, V_2_AlC cannot be exfoliated in KF + HCl solution at 90 °C for 72 h.

From the above results, V_2_AlC can only be effectively exfoliated in NaF + HCl solution. In the three etching solutions, the type and concentration of anions (F^−^ and Cl^−^) are the same and the concentration of cations is also the same. The only difference is the type of cation. Therefore, the type of cation is an important factor that affects the synthesis of V_2_C MXene. For the radius of the three cations, K^+^ > Na^+^ > Li^+^. However, in aqueous etching solution, the cations are hydrated. For the radius of hydrated cations, [Li(H_2_O)_x_]^+^ > [Na(H_2_O)_y_]^+^ > [K(H_2_O)_z_]^+^ [26]. Thus, [Li(H_2_O)_x_]^+^, among the three hydrated cations, should have the largest ability to exfoliate V_2_C MXene. However, LiF is hardly soluble in water, so the concentration of [Li(H_2_O)_x_]^+^ is the smallest, which can be confirmed by the LiF peaks in the XRD of Figure 1. Thus, LiF + HCl can be used to exfoliate V_2_AlC; however, the exfoliation is not thorough. For [Na(H_2_O)_y_]^+^, the radius is larger than that of [K(H_2_O)_z_]^+^ and the concentration is much larger than that of [Li(H_2_O)_x_]^+^. Therefore, [Na(H_2_O)_y_]^+^ can be used to thoroughly exfoliate V_2_AlC, and NaF + HCl is the best etching solution for making V_2_C MXene.

Initially, MXenes (Ti_3_C_2_, Ti_2_C, etc.) were synthesized in HF solution [1,27]. Thus, H^+^ and F^−^ are considered to be essential for the synthesis of MXene. In order to reduce the toxicity and causticity of HF, fluoride salt (LiF/NaF/KF, etc.) + HCl was used to replace HF [21,28]. However, in this research, it is found that, for the V_2_C synthesis, NaF + HCl not only reduces the toxicity of HF, but also increases the exfoliating ability by providing Na^+^. Besides Na^+^, compared with HF, NaF + HCl also provides Cl^−^. Does the existence of Cl^−^ affect the synthesis? In order to investigate the influence of Cl^−^, we conducted the following experiment: 1.44 g V_2_AlC powders were soaked in NaF + HF solution without Cl^−^. This NaF + HF solution was composed of 1 mL HF (48 mol/L, Aladdin Co., Shanghai, China), 99 mL distilled water, and 0.50 g NaF. The F^−^ concentration was 0.6 mol/L, consistent with the NaF + HCl solution (0.6 mol/L). As shown in the top pattern of Figure 1, the main composition of the etched product in NaF + HF was still V_2_AlC. Thus, as is the same as the effect of Na^+^, H^+^, and F^−^, the existence of Cl^−^ in etching solution is essential for the synthesis of highly pure V_2_C MXene.

At this point, we do not clearly know the chemical reason why Cl^−^ is required in the etching. A possible reason is that the concentration of Na^+^ is important for the exfoliation of V_2_AlC. NaF has a better solubility in NaF + HCl solution than that in NaF + HF solution due to the concentration of F^−^. Thus, NaF + HCl has a better exfoliation ability than NaF + HF.

Table 2 lists the 2θ (°) of the (002) peak of newly formed V_2_C MXene by different methods and the intensity ratio of V_2_C’s (002) peak to V_2_AlC’s (002) peak (*I_MXene_/I_MAX_*). The *I_MXene_/I_MAX_* for V_2_C MXene etched by NaF + HCl is 18.11, and this value is much higher than the value (1.00 or 0.22) of previously reported samples etched by HF at room temperature [4,19]. This means that the V_2_C MXenes made by this method were much purer than the samples reported in previous literature.

**Figure 2:**
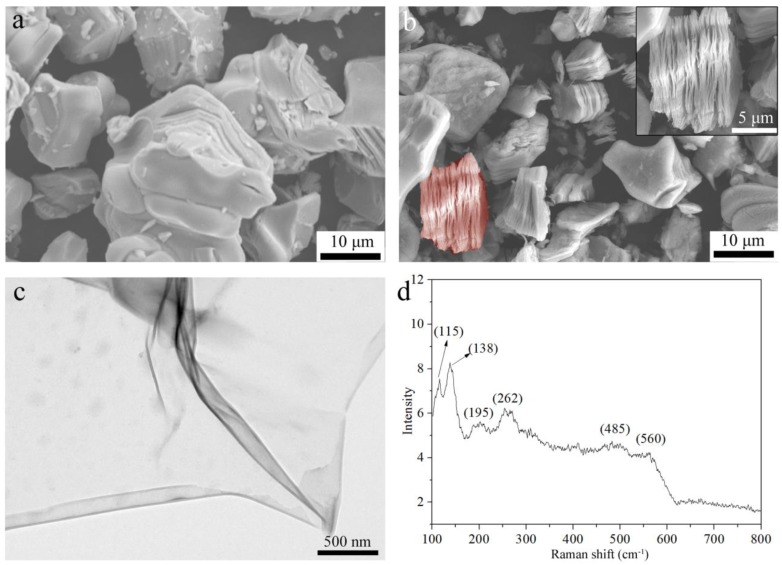
SEM (**a**,**b**) and TEM (**c**) images and Raman spectrum (**d**) of samples. (**a)** V_2_AlC, (**b**–**d)** exfoliated product in NaF + HCl. The inset is an enlarged image of (**b**).

Figure 2a,b show the SEM images of V_2_AlC and etched samples. As shown in Figure 2a, V_2_AlC exhibits typical dense ceramic particles. As shown in Figure 2b, etched samples by NaF + HCl have a multi-layer stacked structure, and the inset has a typical 2D stack structure of MXene. Thus, exfoliation was achieved and quasi-2D MXene sheets were obtained by NaF + HCl etching. All the SEM results in Figure 2a,b agree well with the conclusions drawn from the XRD results in Figure 1.

In order to determine the element content on the sample surface, the sample was analyzed by EDS. The EDS results of V_2_AlC after being treated at 90 °C for 72 h in NaF and HCl solution indicated the presence of V, C, O, F, and small amounts of Al at an atomic ratio of 2.00:1.00:0.98:0.3:0.03, respectively. Assuming, conservatively, that the entirety of the Al signal originates from unreacted V_2_AlC, the amount of Al would be around 0.59 wt.% after treatment. This indicates that only a very small part of V_2_AlC had not been etched. This is more proof that highly pure V_2_C MXene was successfully prepared.

Figure 2c is the TEM image of a piece of fully exfoliated V_2_C sheet. From this image, the electron-transparent thin morphology and 2D structure of V_2_C MXene are shown. Moreover, the thin nanosheet is presented as flexible. Figure 2d is the Raman spectrum of V_2_C MXene. The bands of V_2_AlC at 158, 240, 257, and 361 cm^−1^ and weaker peaks near 413, 509, and 693 cm^−1^ [29,30] vanished and new bands appeared at 115, 138, 195, 262, 485, and 560 cm^−1^. According to the theoretical work of Champagne et al. [30], the peak at 195 cm^−1^ corresponds to the E_g_ model of V_2_CF_2_; the peak at 262 cm^−1^ corresponds to the E_g_ model of V_2_C; the peak at 485 cm^−1^ corresponds to the E_g_ model of V_2_C(OH)_2_; and the peak at 560 corresponds to the A_1g_ model of V_2_CF_2_ and V_2_C(OH)_2_. The E_g_ model is due to the in-plane vibration of V atoms and the A_1g_ model is due to the out-of-plane vibration of V atoms. The two peaks at 115 and 138 cm^−1^ cannot currently be explained. Champagne et al. [30] reported similar results where, in the spectrum of V_2_C MXene, sharp peaks of V_2_AlC vanished. However, only two unsharp peaks at ~260 and ~410 cm^−1^ appeared in the spectrum. Thus, the spectrum in Figure 2d has more peaks that fit the theoretical prediction in [30]. The difference should be because the V_2_C MXene samples were made by different methods (NaF + HCl high temperature etching in this paper and HF room temperature etching in [30]) and the samples had different terminations and impurities.

### 3.2. Thermal Stability Analysis

**Figure 3:**
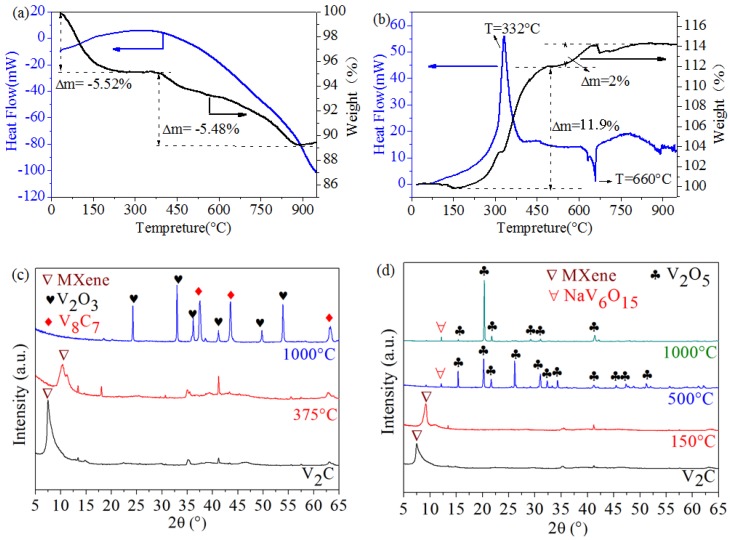
TG and DTA curves of V_2_C MXene in (**a**) Ar atmosphere and (**b**) air atmosphere. XRD patterns of V_2_C MXene samples after heat treatment in (**c)** Ar atmosphere and (**d**) air atmosphere.

**Figure 4:**
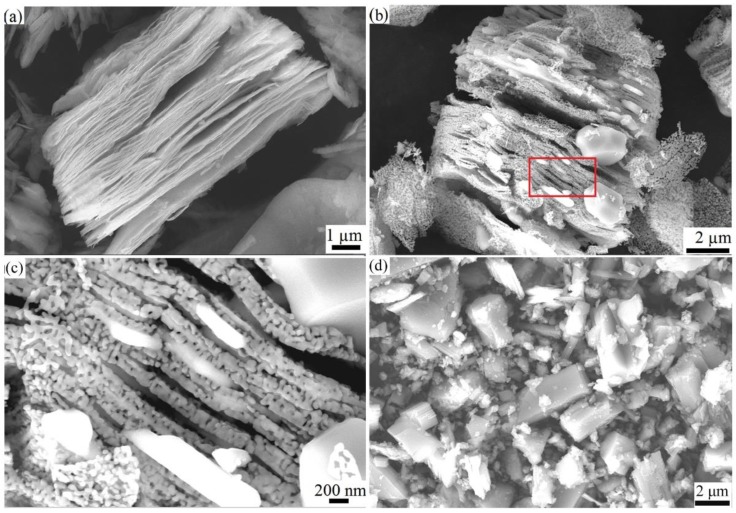
SEM images of V_2_C MXene after heat treatment at (**a**) 150 °C in Ar; (**b**) 1000 °C in Ar; (**c**) is a highly magnification image of area in the red rectangle of b; (**d**) at 1000 °C in air.

The TG curves and DTA curves of as-prepared V_2_C MXene etched by NaF + HCl are shown in Figure 3a,b. In order to know the phase and morphology change at a high temperature, V_2_C MXene samples were heat treated at different temperatures in Ar or air atmosphere. The XRD patterns of the sample before and after heat treatment are shown in Figure 3c,d. SEM micrographs are shown in Figure 4. Based on these results, the phase change of V_2_C MXene from room temperature (RT) to 1000 °C is discussed.

#### (1) Thermal Stability in Ar

In Ar atmosphere, the TG curve of V_2_C MXene in Figure 3a is divided into two stages. The first stage is 5.52% weight loss from RT to 375 °C. Most of the weight loss occurred in the temperature range of 70–120 °C, which is due to the loss of physically adsorbed water. The DTA curve at this temperature range does not display obvious change. Additionally, the structure of MXene has not changed. The second stage is 5.48% weight loss from 375 °C to 900 °C, which is caused by the loss of OH/O/F terminated groups. The weight loss needs energy to break the bonds between OH/O/F and V_2_C crystals. Thus, the corresponding DTA curve is obviously endothermic. In this process, OH/O/F are released in the form of H_2_O/O_2_/HF. At high temperatures (>900 °C), almost all terminated groups have already been lost; therefore, the weight loss stops. At such a high temperature, V_2_C reacts with previously released oxygen to form oxides. Thus, there is a weak weight gain. According to this analysis, the heat process of V_2_C in Ar is mainly a weight loss process at a low temperature and a weak weight gain at a temperature >900 °C.

The conclusion drawn from Figure 3a can be supported by the XRD patterns in Figure 3c and the SEM micrographs in Figure 4. In Figure 3c, after heating at 375 °C in Ar, the sample’s main composition was still V_2_C MXene. The physically adsorbed water and some surface terminations were lost, and the interlayer distance of V_2_C MXene was reduced. As shown in Figure 3c, the 2θ angle of the (002) peak shifted from 9.172° to 10.35°, which means that the *d* space of the basal plane, including the thickness of the V_2_C layer and interlayer distance, was reduced from 0.963 nm to 0.854 nm. This reduction of the value was due to the reduction of the interlayer distance. After heat treatment at 1000 °C, some V_2_C was oxidized to form V_2_O_3_, and some V_2_C was transformed to V_8_C_7_.

From the SEM image in Figure 4a, 150 °C treatment did not change the 2D structure of V_2_C MXene. From Figure 4b,c, however, 1000 °C treatment obviously changed the 2D structure of V_2_C MXene. A large number of nanoparticles appeared and were distributed on the surface and in the interlayer space of the multilayered 2D structure. From EDS, the particles consisted of V and O elements, and the element ratio was 2.1:2.9, so it can be confirmed that these particles were V_2_O_3_. This result agrees with the XRD patterns in Figure 3c.

The derived material consisted of V_2_O_3_ nanoparticles distributed in the 2D structure. The vanadium oxide had a very high specific surface area. Vanadium oxide is used as a catalyst in many areas. If the composition and structure can be better controlled, the derived material can be applied as a catalyst with a high performance.

#### (2) Thermal Stability in Air

In air atmosphere, as shown in Figure 3b, the first stage of the TG curve shows a weak weight loss (RT to 150 °C) due to the loss of physically adsorbed water. As shown in Figure 3d, the XRD pattern of V_2_C does not display obvious change, except for the fact that the (002) peak shifts to a high angles direction.

With the temperature rising, the surface of V_2_C begins to be oxidized in air atmosphere. Therefore, there is a large exothermic peak at 332 °C in the DTA curve and a drastic weight gain in the TG curve (Figure 3b). According to the XRD pattern in Figure 3d, the oxidation product is V_2_O_5_ rather than V_2_O_3_, because there is enough oxygen in air atmosphere. After the exothermic peak at 332 °C, there is an endothermic peak at ~660 °C, which corresponds the melt of newly formed V_2_O_5_ (the melting point of pure V_2_O_5_ is 690 °C [31]). Therefore, there is an exothermic peak (332 °C) and an endothermic peak (660 °C) in the DTA curve of V_2_C.

According to the XRD and SEM results in Figure 3d and Figure 4d, the oxidation products of V_2_CT_x_ in air at 1000 °C are a lot of V_2_O_5_ crystals in a rectangular block shape and a small amount of NaV_6_O_15_ [32].

The following reaction is proposed to describe the oxidation of V_2_CT_x_ in air:

V_2_CT_x_ + 3.5 O_2_ = V_2_O_5_ + CO_2_↑ + T_x_↑

## 4. Conclusions

Based on the research in this paper, it is concluded that only NaF + HCl etching solution can be used to synthesize highly pure V_2_C MXene. Neither LiF + HCl nor KF + HCl can be used. The existence of Na^+^ and Cl^−^ in etching solution is essential for the synthesis of highly pure V_2_C. The chemical reason for this is related to the radius of hydrated cations and the solubility fluoride salts. From the radius of hydrated cations, Na^+^ > K^+^; from the solubility, NaF > LiF. Thus, Na^+^ has a better exfoliation ability than Li^+^ and K^+^. Additionally, NaF has a better solubility in NaF + HCl solution than in NaF + HF solution. Thus, NaF + HCl has a better exfoliation ability than NaF + HF. The suitable condition is etching in NaF + HCl solution at 90 °C for 72 h.

The synthesized V_2_C MXene was stable in Ar atmosphere below 375 °C; above this temperature, V_2_C was oxidized to form V_2_O_3_ nano-crystals, which were evenly distributed on 2D V_2_C sheets. In air, V_2_C maintained a stable 2D structure at 150 °C; above this temperature, V_2_C was oxidized and V_2_O_5_ was the final oxidized product.

## Figures and Tables

**Table 1:** Abbreviations and the corresponding full names of chemical substances.

Abbreviation	Full Name
V_2_C MXene	two-dimensional vanadium carbide
V_2_AlC	vanadium aluminum carbide
V_2_O_3_	vanadium trioxide
V_2_O_5_	vanadium pentoxide
V_8_C_7_	vanadium carbide
NaV_6_O_15_	sodium vanadium oxide
LiF	lithium fluoride
NaF	sodium fluoride
KF	potassium fluoride
HCl	hydrochloric acid
HF	hydrofluoric acid
NaCl	sodium chloride

**Table 2:** 2θ (°) of the (002) peak of newly formed V_2_C by different methods and the intensity ratio of V_2_C’s (002) peak to V_2_AlC’s (002) peak (*I_MXene_/I_MAX_*).

Etching Solution	2θ (°) of (002) Peak	*I_MXene_/I_MAX_*
LiF + HCl	9.13	0.66
NaF + HCl	8.03	18.11
KF + HCl	-	-
NaF + HF	7.8	0.18
40% HF [19]	7.33	1.00
50% HF [4]	8.96	0.22
